# The Epidemiology of Coccidioidomycosis (Valley fever) and the Disease Ecology of *Coccidioides* spp. in New Mexico (2006–2023)

**DOI:** 10.3390/pathogens14060607

**Published:** 2025-06-19

**Authors:** Paris S. Salazar-Hamm, Sarah Shrum Davis, Jovani Catalán-Dibene, Adriana L. Romero-Olivares, Karen Edge, Andrew W. Bartlow, Donald O. Natvig, Morgan E. Gorris

**Affiliations:** 1 Department of Biology, University of New Mexico, Albuquerque, NM 87106, USA; psh102@unm.edu (P.S.S.-H.); dnatvig@unm.edu (D.O.N.); 2 Department of Biology, New Mexico Institute of Mining and Technology, Socorro, NM 87801, USA; 3 Infectious Disease Bureau, New Mexico Department of Health, Santa Fe, NM 87505, USA; 4 Emerging Infections Program, Office for Community Health, University of New Mexico, Albuquerque, NM 87131, USA; 5 Department of Biology, New Mexico State University, Las Cruces, NM 88003, USA; jcatalan@nmsu.edu (J.C.-D.); alro@nmsu.edu (A.L.R.-O.); 6 Genomics and Bioanalytics, Los Alamos National Laboratory, Los Alamos, NM 87545, USA; abartlow@lanl.gov; 7 Information Systems and Modeling, Los Alamos National Laboratory, Los Alamos, NM 87545, USA

**Keywords:** *Coccidioides*, coccidioidomycosis, Valley fever, mycoses, climate change, disease surveillance, qPCR, burrow, soil

## Abstract

Coccidioidomycosis (Valley fever), caused by *Coccidioides* spp., is a fungal infection endemic to semi-arid regions of the Americas. Despite 80 years of disease recognition in New Mexico, there is limited disease awareness. We incorporated clinical, epidemiological, and ecological datasets to summarize the knowledge of Valley fever in New Mexico. We analyzed 1541 human cases from 2006 to 2023. On average, 86 cases were reported each year (4.1 cases per 100,000 population per year). The highest levels of incidence were in southwestern New Mexico. American Indian or Alaska Natives in New Mexico had a 1.9 times higher incidence rate of coccidioidomycosis than White people, and among age groups, older populations in New Mexico had the highest incidence rates. We analyzed 300 soil samples near Las Cruces, New Mexico, for the presence of *Coccidioides* and reported the first known positive soil samples collected from the state, the majority of which were from grassland-dominated sites and from animal burrows. Sequence analyses in clinical specimens, wild animals, and soil samples confirmed that *Coccidioides posadasii* is the main causative species of coccidioidomycosis in New Mexico. Environmental surveillance validated that locally acquired infections could occur in, but are not limited to, Catron, Doña Ana, Sierra, and Socorro Counties.

## 1. Introduction

Though numerous lines of evidence suggest coccidioidomycosis (Valley fever) is endemic to New Mexico, this evidence contrasts with the relatively few reported clinical cases each year. This discrepancy may exacerbate a speculative claim that New Mexico is less endemic than other states, including its westerly neighbor, Arizona. Research dating back to the 1930s first characterized coccidioidomycosis as a fungal infection endemic to the San Joaquin Valley of California [1]. Endemic regions were broadly defined as semi-arid-to-arid climates at low elevations, with alkaline soils. By the 1940s, New Mexico was already among the recognized endemic states [2]. Further support for endemicity in New Mexico came from World War II military recruits from the southwestern US who were tested with the antigen coccidioidin [3]. The results of these skin tests indicated prior exposure to *Coccidioides* environmental spores. Omitting recruits with a history of travel, those with a positive reaction were mapped onto their county of residence. The resultant map highlighted 24 of the 33 New Mexico counties (72.7%) of New Mexico as endemic, all regions except the northeastern corner of the state. In the 1950s, more granular geographic descriptions for *Coccidioides* named the New Mexican cities of Lordsburg (Hidalgo County), Deming (Luna County), Socorro (Socorro County), Alamogordo (Otero County), Roswell (Chaves County), and Carlsbad (Eddy County) as suitable habitats [4].

Following improvements to diagnostics and recognition, coccidioidomycosis became a nationally notifiable disease in 1995 and a reportable disease in New Mexico in 2006. It was only in 2019, 13 years later, that the first clinical sequences from the state were analyzed [5]. In 2022, *Coccidioides* was validated in the environment of New Mexico through a survey of small-mammal lungs [6].

Despite over 80 years of acknowledging New Mexico as an endemic state for coccidioidomycosis, research on coccidioidomycosis and *Coccidioides* in New Mexico has been limited. An advanced PubMed search (accessed on 7 February 2025) revealed only 22 of 6533 publications (0.3%) that contained “coccidioidomycosis”, “Valley fever”, or “*Coccidioides*” in the title or abstract also contained “New Mexico”; in half of these articles, New Mexico was only mentioned anecdotally as part of the endemic range. In response, scientists from across the state have collaborated on a comprehensive investigation of coccidioidomycosis in New Mexico and a unified call for future research. Here, we describe the epidemiology of coccidioidomycosis in New Mexico using case data from the New Mexico Department of Health (NMDOH) from 2006 to 2023. We captured sequence data from human isolates to further describe the clinical landscape of causative agents. Additionally, we provide the first detections of *Coccidioides* in New Mexico soils. The findings from our analyses will provide a baseline to inform future enhanced epidemiologic surveillance and soil surveillance for coccidioidomycosis and *Coccidioides* in New Mexico.

## 2. Materials and Methods

### 2.1. New Mexico Department of Health (NMDOH)’s Epidemiological Data

Coccidioidomycosis is a reportable condition in New Mexico per administrative code 7.4.3.13, and data are available starting in 2006. The NMDOH follows the Council of State and Territorial Epidemiologists (CSTE)’s case definitions [7], requiring both laboratory and clinical evidence of disease. It should be noted that there were revisions to the case definitions in 2008, 2011, and 2023 [7,8], potentially introducing bias in comparisons between case counts over different time periods. New Mexico is considered a low-incidence jurisdiction (the average coccidioidomycosis incidence is ≤10 confirmed cases per 100,000 population per year for a period of three consecutive years) by the CSTE and Centers for Disease Control and Prevention (CDC) for case classification purposes [7]. In a low-incidence jurisdiction, cases are considered confirmed when a case has either (1) confirmatory laboratory evidence and has either epidemiologic linkage or clinical criteria, or (2) presumptive laboratory evidence and has both epidemiologic linkage and clinical criteria. Cases are probable when they have either (1) confirmatory laboratory evidence, but do not meet epidemiologic linkage or clinical criteria, or (2) presumptive laboratory evidence and either epidemiologic linkage or clinical criteria. Cases are suspect when there is presumptive laboratory evidence for coccidioidomycosis infection, but there is no epidemiologic linkage or sufficient clinical criteria. Cases are geographically classified by county of residence. Routine reporting also collects data on basic demographics, such as sex, age, race, and ethnicity. Cases are investigated by the NMDOH via medical record review for clinical characteristics such as disease onset and symptoms, but patient interviews are not routinely conducted. The major limitations of medical record review are that domestic travel, historically remote travel, occupation, and potential exposures may not be captured.

We analyzed 1541 cases of coccidioidomycosis investigated by medical record review from 2006 to 2023. We reported demographic information, the time series data of case counts (monthly and yearly), and the incidence by county. To calculate disease incidence rates at the county level, we used county population estimates from the US Census Bureau’s census and intercensal population estimates [9,10,11] (Supplementary Figure S1). To calculate case and incidence rates among sex, race, ethnicity, and age groups, we used state-level demographic information from the US Census Bureau’s American Community Survey (ACS) 1-Year Estimates, 2006–2023 [12], gathered using tidycensus v.1.4.4 in R [13]. ACS data were unavailable for the year 2020, so 2019 data were imputed. We calculated the incidence rate ratios and 95% confidence intervals for select demographics.

### 2.2. Molecular and Phylogenetic Analyses of Clinical Coccidioides

Clinical specimens from 14 patients diagnosed with coccidioidomycosis in New Mexico between 2018 and 2023 were submitted to the NMDOH’s Scientific Laboratory Division (SLD). Two specimens were obtained from one patient, and the remaining had one specimen each. NMDOH’s SLD extracted *Coccidioides* DNA from 15 human specimens using the PrepMan^®^ Ultra Reagent method (Applied Biosystems, Foster City, CA, USA), and samples were stored at −80 °C until a subsample was transferred to the Natvig Laboratory at the University of New Mexico. We amplified the internal-transcribed spacer region (ITS) of the ribosomal RNA (rRNA) with the ITS1-F and ITS4 primers [14,15]. We performed polymerase chain reactions (PCRs) with an initial step at 95 °C for 5 min, followed by 35 cycles at 94 °C for 30 s, 50 °C for 30 s, and 72 °C for 45 s, before a final extension at 72 °C for 7 min. Crude PCR products were sent to Functional Biosciences (Madison, WI, USA) for Sanger sequencing using BigDye v.3.1 chemistry and an ABI 3730xl DNA analyzer (Applied Biosystems, Foster City, CA, USA). The sequence quality was assessed with a minimum phred20 cutoff. The forward and reverse sequences were visualized and assembled with Sequencher v.5.1 (Gene Codes, Ann Arbor, MI, USA). We deposited the sequences in GenBank under PQ901548–PQ901562 (Supplementary Table S1).

The ITS rRNA sequences collected from patients in New Mexico were subjected to phylogenetic analysis along with the reference sequence *Uncinocarpus reesii* from GenBank (accession NR_111092) [16]. We aligned the sequences with mafft v.7.481 using the default settings [17] and trimmed the resulting alignment with trimal v.1.4.1 in automated1 mode [18]. A maximum likelihood phylogeny for within-state comparisons was inferred using the TNe model from 33 *Coccidioides* sequences from New Mexico, plus *U. reesii* as an outgroup, from a final alignment of 583 nucleotides. The best-fitting model for tree building was chosen by the ModelFinder algorithm [19]. The tree was constructed in IQ-Tree v.1.6.12 [20], with 10,000 ultrafast bootstrap replicates, and visualized in ggtree [21]. The alignment and tree file were deposited in Dryad (https://doi.org/10.5061/dryad.sn02v6xgg).

### 2.3. Molecular Identification of Coccidioides in Soils

In May 2023, a summer surveillance effort was set up at the Jornada Experimental Range in Doña Ana County, New Mexico. We sampled three sites, each with a different type of vegetation: grassland-dominated (predominantly black grama [*Bouteloua eriopoda*]), ecotone (i.e., the transition between grassland and shrubland), and shrubland-dominated (honey mesquite [*Prosopis glandulosa*]). At each of the three sites, we localized ten burrows and set up flags for identification. We proceeded to sample approximately 10 g of soil from 5–10 cm into the burrow and another 10 g of soil 5–10 cm to the side of the opening of the burrow (i.e., topsoil). We sampled these microsites repeatedly, five times throughout 8 weeks, on 26 May, 13 June, 27 June, 11 July, and 25 July of 2023. In total, we collected 300 samples. Samples were kept in a cooler for 1–2 h between the time of collection and arrival at the lab, where they were frozen and kept at −80 °C for 24–48 h until processed.

We extracted DNA from soil samples using the DNeasy PowerSoil Pro Kit (Qiagen, Hilden, Germany) and tested for the presence of *Coccidioides* using the CocciDx assay, which is better at identifying *C. posadasii* in the soil compared with CocciEnv, which is better for detecting *C. immitis* (personal communication with D.S. Kollath). We tested each sample in triplicate following the recommendations for the CocciDx assay in the study by Bowers and collaborators [22]. Briefly, each 10 uL reaction had a 1X TaqMan Environmental Master Mix (Applied biosystems, Foster City, CA, USA), 1X of Custom TaqMan MGB probe (ThermoFisher rScientific, Waltham, MA, USA), 1X of each primer, and approximately 50 ng of the DNA template. We tested the samples using a BioRad CFX Connect thermal cycler with an initial denaturation step at 95 °C for 10 min, followed by 40 cycles at 95 °C for 15 s and 60 °C for 1 min. We considered samples as positive when at least two out of three samples had cycle threshold (CT) values of ≤40, with logarithmic amplification, and consistent controls (positive, negative, and blank). As a positive control, we used DNA from *C. posadasii* Δchs5; as a negative control, we used DNA from *Phoma* spp.; and as a blank, we used water.

To infer species from qPCR-positive samples, we targeted a 372-nucleotide mitochondrial intron sequence of the *cytochrome c oxidase subunit 1* (*cox1*) gene that is present in *C. posadasii* but not *C. immitis*. We amplified this region with the P2F (5′-TCAAATCATGTGTAATATGTGG-3′) and P2R (5′-GTTGACCATAAAGAAAAGTTGG-3′) primer pair [5], followed by 2% gel electrophoresis, and Sanger sequencing using BigDye v3.1 (Applied Biosystems, Foster City, CA, USA) chain termination with the Big Dye STeP protocol [23]. The sequences were deposited in GenBank under accession numbers PV177154–PV177156 (Supplementary Table S2).

## 3. Results

### 3.1. Epidemiology of Coccidioidomycosis in New Mexico

**Figure 1:**
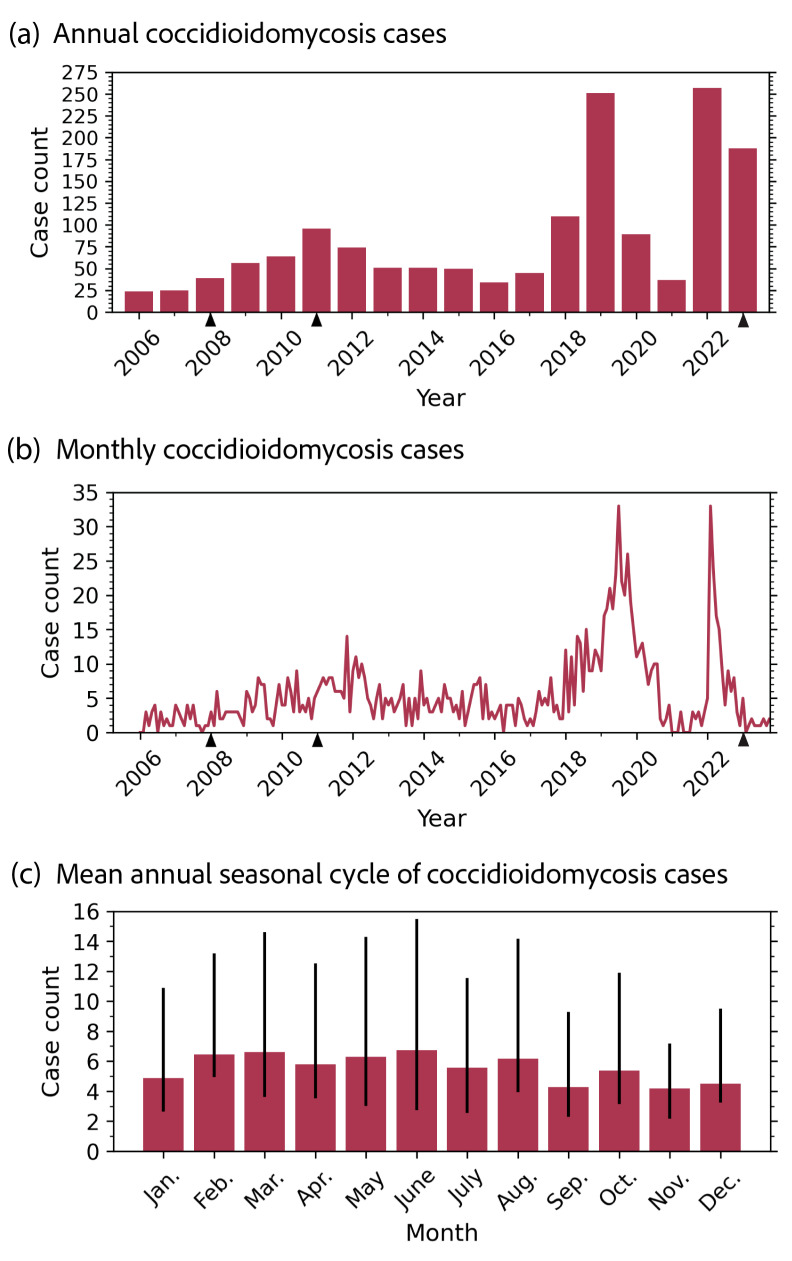
Time series of (**a**) annual coccidioidomycosis cases and (**b**) monthly coccidioidomycosis cases in New Mexico from 2006 to 2023. Coccidioidomycosis case definitions changed in 2008, 2011, and 2023; these years are indicated by black arrow markers on the x-axis. (**c**) Mean annual seasonal cycle and the interquartile range of coccidioidomycosis cases in New Mexico from 2006 to 2023. The greatest case seasonal counts and variability occurred in the spring and summer seasons.

We analyzed 1541 cases of coccidioidomycosis reported in New Mexico (2006–2023 population: 2,074,480) over a 17-year period (2006–2023) investigated by the NMDOH via a medical record review. On average, 86 coccidioidomycosis cases were reported in New Mexico each year (range: 24–257 cases; Figure 1a), with a mean annual state incidence of 4.1 cases per 100,000 population per year (range: 1.2–12.2 cases per 100,000 population per year). Based on CSTE definitions, 946 (61.4%) cases were confirmed, 56 (3.6%) were probable, and 539 (35.0%) were suspect (Table 1).

Between the first five years of reporting (2006–2010) and the last five years of our dataset (2019–2023), coccidioidomycosis cases in New Mexico statistically significantly increased from an average of 42 cases per year to 164 cases per year (Welch’s *t*-test; *p* = 0.05). This was an increase of 295% (Figure 1a). The years of 2019, 2022, and 2023 were all exceptionally high for case reports, reporting 251, 257, and 188 cases, respectively. There was also a sharp decrease in cases observed from 2019 to 2020–2021. The monthly case counts suggest a fair amount of interannual variability in when cases were reported, though the relatively few cases (<35 cases) reported each month may have increased the noise in the time series (Figure 1b). The mean annual seasonal cycle of coccidioidomycosis cases from 2006 to 2023 did not suggest a strong seasonal trend (Figure 1c). Cases were relatively lower in late summer to midwinter (September–January) compared with late winter through midsummer (February–August), though the difference in the mean incidence between these seasons was not statistically significant (Welch’s *t*-test; *p* = 0.75). The standard deviation for each month indicates that the greatest case variability generally occurred in the late winter to midsummer (February–August), too.

**Figure 2:**
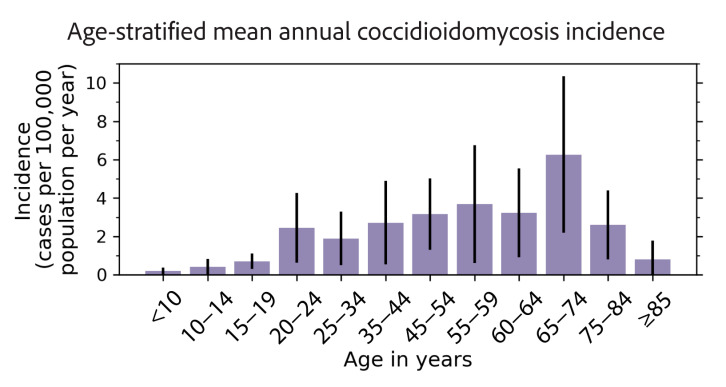
Age-stratified mean annual coccidioidomycosis incidence rates in New Mexico, averaged for 2006–2023, and 95% confidence intervals. Older populations had higher levels of coccidioidomycosis incidence, with the highest incidence among people aged 65–74 years.

Most coccidioidomycosis case-patients were adults (median: 55 years; range: 1–92 years; Table 1). Less than 4% of cases were pediatric (<18 years), and 28.6% were geriatric (≥65 years old). The crude coccidioidomycosis incidence rate stratified by age suggests older populations in New Mexico were more affected by this disease (Figure 2). Apart from two outlier age groups (20–24; 65–74), the incidence increased with age until peaking in case-patients aged 55–59 years (3.7 cases per 100,000 population per year), then decreased at 60 years and older. The coccidioidomycosis incidence was highest among case-patients aged 65–74 years (6.3 cases per 100,000 population per year). The lowest incidence rate was among case-patients aged under 10 years (0.2 cases per 100,000 population per year). However, the large 95% confidence intervals illustrate that there was a high degree of uncertainty around these means. Males accounted for 57.4% of cases (Table 1) and were more likely to contract coccidioidomycosis compared with females in New Mexico (Table 2).

Regarding ethnicity and race, non-Hispanic or Latino people accounted for 59.2% of cases but did not have a statistically significantly higher incidence rate than Hispanic or Latino people (Table 1 and Table 2). The incidence rate of coccidioidomycosis among people who identified as American Indian or Alaska Native in New Mexico was 1.9 times higher than in White people (Table 2). We did not find a significantly higher incidence rate in people who identified as Black or African American in New Mexico compared with the referent White population. Only nine case-patients identified as Asian.

The median time between illness onset and diagnosis was 10 days (740 cases; range: 0–2935 days; IQR: 3–32 days). Of 631 cases with outcome information, 438 (69.4%) were admitted to the hospital, and the median length of stay was 6 days (IQR: 3–11 days; Table 1). A total of 20 (6.4%) fatalities were reported (Table 1). There was limited data available (<200 case-patients) regarding case-patient work-up, symptoms, comorbidities, treatment, occupation, and travel information.

**Figure 3:**
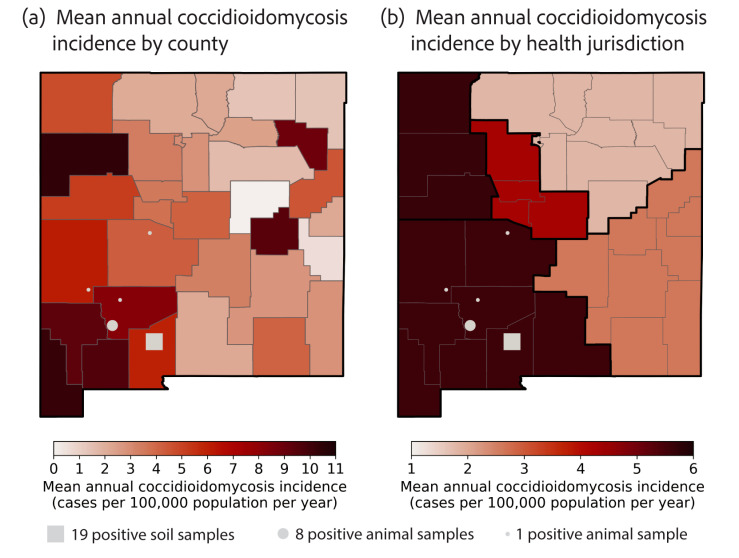
(**a**) Mean annual coccidioidomycosis incidence (cases per 100,000 population per year) averaged from 2006 to 2023 by county. (**b**) Mean annual coccidioidomycosis incidence (cases per 100,000 population per year) averaged from 2006 to 2023 by New Mexico Health Jurisdiction (Supplementary Figure S1). In both maps, positive soil samples collected in 2023 and animal samples collected in 1998, 2014, and 2017 are indicated with grey markers, positive soil samples with squares, and positive animal samples with circles. The size of the marker corresponds to the number of positive samples within a given 12 km diameter surrounding a sampling location.

The county-level mean annual incidence averaged from 2006 to 2023 ranged from 0 to 10.4 cases per 100,000 population per year (Figure 3a; Supplementary Figure S1). The counties with the highest mean annual incidence were McKinley (10.4 cases per 100,000 population per year), Hidalgo (10.2 cases per 100,000 population per year), Luna (9.6 cases per 100,000 population per year), De Baca (9.4 cases per 100,000 population per year), and Grant (9.3 cases per 100,000 population per year). Generally, counties in the western half of the state had a higher incidence than the eastern half of the state, and counties in southwestern New Mexico had the highest incidence (Figure 3a). Two counties in the eastern half, Harding and De Baca, also had high levels of mean annual incidence (8.9 and 9.4 cases per 100,000 population, respectively); however, these incidence levels were driven by few (<5) cases in the counties and very low populations (2006–2023 Harding County population: 682, De Baca County population: 1864; Supplementary Figure S1).

We also explored geographic trends across the five primary health jurisdictions in New Mexico: Metro, Northeast, Northwest, Southeast, and Southwest (Supplementary Figure S1). Our patient-case data had jurisdiction information coded from patient residences for 1539 cases. The Metro jurisdiction had the highest number of cases (*n* = 696), followed by the Southwest (*n* = 372) and Northwest (*n* = 228; Table 3). Of the cases in the Northwest jurisdiction, 27 were attributed to the Northwest Tribal designation, which most likely refers to the Navajo Nation. An additional ten cases were associated with tribes but were not partitioned into a jurisdiction. Two cases were identified as being from out of state. After accounting for population, the Southwest (5.6 cases per 100,000 per year) and Northwest (5.6 cases per 100,000 per year) jurisdictions had the highest mean annual coccidioidomycosis incidences (Figure 3b; Table 3).

### 3.2. Clinical Coccidioidomycosis Sequence Analyses

We analyzed 15 clinical specimens from 14 patients diagnosed with coccidioidomycosis in New Mexico between 2018 and 2023 (Supplementary Table S1). Patient data were collected by the NMDOH through passive surveillance. Thus, information availability was dependent on healthcare providers’ thoroughness, and many data fields were, unfortunately, unknown. Seven patients were male, and five were female (two were unknown). Patients’ ages ranged between 25 and 88 years. Race and ethnicity data were available for nine patients (five were unknown), of which eight were White and Hispanic, and one was White and non-Hispanic. Eight of the patients presented with a pulmonary infection (six were unknown). Specimens were mainly collected from sputum (*n* = 6), but there was one specimen each from pleural effusion, tissue, bronchial wash, body fluid, and an abscess (three were unknown). Two patients had significant past medical histories (eleven were unknown), including a smoker with type II diabetes and a patient with a fungal co-infection by *Pichia kudriavzevii* (clinically referred to as *Candida krusei*). Two patients were suspected of travel-acquired coccidioidomycosis due to their travel or residential history in Arizona and California; all others were unknown. Occupational data were only available for one patient, who reported working in outdoor construction. The taxonomic identification of the clinical specimens using ITS rRNA indicated the disease was caused by *Coccidioides posadasii* in 13 cases and *Coccidioides immitis* in 1 case. The case of *C. immitis* was the patient who previously resided in California.

**Figure 4:**
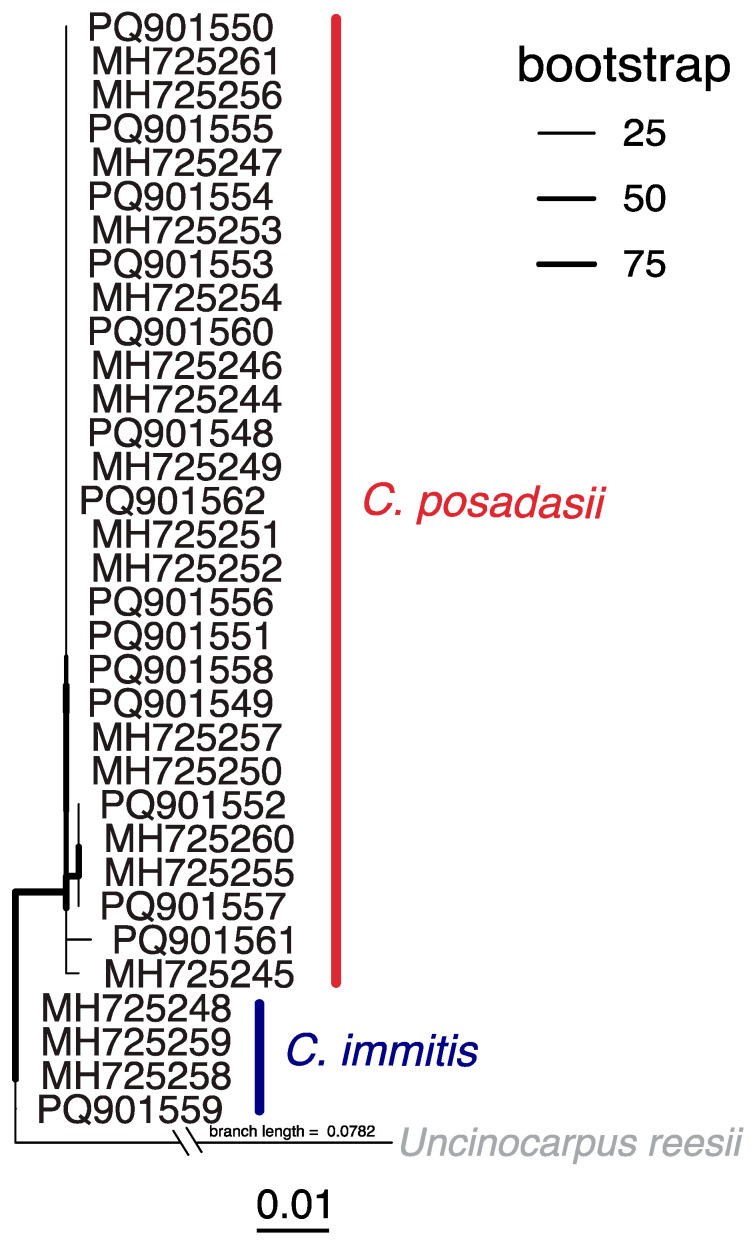
Maximum likelihood (ML) phylogenetic analysis of 33 *Coccidioides* ITS rRNA sequences from New Mexico. ML tree was inferred using best-fitting model, as determined by ModelFinder, with 10,000 ultrafast bootstrap replicates (denoted by branch thickness). The two described causative agents of coccidioidomycosis, *C. immitis* (dark blue) and *C. posadasii* (dark red), are included. Branch length of outgroup *Uncinocarpus reesii* (GenBank accession NR_111092) is 0.0782.

A total of 33 ITS rRNA sequences from patients diagnosed in New Mexico reported here (*n* = 15) and previously by Hamm and colleagues [5] revealed coccidioidomycosis cases were caused by both *C. posadasii* (*n* = 29) and *C. immitis* (*n* = 4), but *C. posadasii* was the dominant causative agent (Figure 4; Supplementary Table S1). *C. immitis* strains from New Mexico were related to those from California, Utah, and Mexico, while *C. posadasii* strains from New Mexico were related to those from patients in Arizona, Texas, Argentina, and Brazil, as well as from travelers from non-endemic regions. There were several clades of clinical sequences from New Mexico that were closely related (Figure 4). For example, 20 of the *C. posadasii* strains were identical (0 single-nucleotide polymorphisms (SNPs)). Few SNPs in the ITS rRNA region and incomplete travel histories contributed to the difficulty in ascertaining which of these infections were acquired locally.

### 3.3. Coccidioides in New Mexico Soils

Between May and July 2023, we surveyed soils in and proximal to animal burrows in three distinct habitat types (i.e., grassland-dominated, mesquite shrubland-dominated, and the ecotone) at the Jornada Experimental Range, about 40 km north of Las Cruces in Doña Ana County, New Mexico. Of 300 samples collected, 19 (6.3% positivity rate) were positive for *Coccidioides* spp. with CocciDx qPCR amplification (Supplementary Table S2). Two positive samples were collected in May (26 May 2023), two in June (27 June 2023), and fifteen in July (11 July and 25 July 2023). Eleven were from animal burrows (57.9% of positive samples; 7.3% positivity rate) and eight were from topsoil (42.1% of positive samples; 5.3% positivity rate). The difference in *Coccidioides* positivity between animal burrows and topsoil was not statistically significant (chi-squared test; *p* = 0.64).

Twelve positive samples (63.2% of total positive samples; 12% positivity rate) were from the grassland-dominated site, half of which were collected from burrows and half from topsoil (Table 4). By pairwise comparison via Fisher’s exact test, *Coccidioides* positivity was statistically significantly higher in the grassland-dominated site than in the ecotone (*p* = 0.05) and mesquite shrubland-dominated sites (*p* = 0.02). There was not a statistically significant difference in *Coccidioides* positivity between the ecotone and mesquite shrubland-dominated sites (*p* = 1.0). Of the 12 positive samples from grasslands, 11 were collected in the last two sampling time points (i.e., July 2023). Interestingly, no microsites tested positive consistently throughout our sampling. That is, the microsites that tested positive in July did not test positive in earlier samplings. Similarly, most positive samples were not paired (i.e., a positive burrow and topsoil at one microsite at the same time point), with the exception of one. Of the ecotone sites, there were four *Coccidioides*-positive samples (21.1% of positive samples; 4% positivity rate), two from burrows and two from topsoil (Table 4). Of the mesquite shrubland sites, we found three *Coccidioides*-positive samples (15.8% of positive samples; 3% positivity rate), all of them from burrows (Table 4).

To infer the species of the positive soil samples, we targeted a mitochondrial intron sequence of the *cytochrome c oxidase subunit 1* (*cox1*) gene that is present in *C. posadasii* but not *C. immitis*. We performed PCR on a subset of samples (*n* = 10), which resulted in confirmation of the *C. posadasii* intron sequence in 5 samples (Supplementary Table S2). Four of the samples for which the intron was amplified were from burrows (C7-B, C10-B, C2-B, and C7-B), and three samples were from the same site (C7-B-Jul11, C7-T, and C7-B-Jul25). We were able to sequence the *cox1* intron segment from three of these samples (Supplementary Table S2). Because all soil samples were taken within 10 km of each other from the grassland-dominated sites, we believe that failure to amplify the fragment was likely indicative of a low fungal load rather than the absence of the intron.

## 4. Discussion

Our epidemiologic analysis of coccidioidomycosis cases in New Mexico suggests increasing disease recognition and/or disease burden of coccidioidomycosis in the state. From the start of the reporting time period (2006–2010) to the last five years (2019–2023), the number of reported cases increased by 295%. According to public health surveillance data reported by the CDC [24], this relative increase in cases was larger than the relative increases in Arizona and California over a similar time period (2006–2010 compared with 2018–2022), though similar to increases in both states over alternative time periods. Cases in Arizona increased by 35% (from 7450 cases to 10,074 cases on average), and cases in California increased by 147% (from 3166 cases to 7831 cases on average). Though New Mexico reported far fewer cases each year than California and Arizona, after accounting for population density, disease incidence suggests it is among the most endemic states in the US. Among states with incidence estimates, Arizona reports ~135 cases per 100,000 population per year [25], California reports ~12 cases per 100,000 population per year [25], New Mexico reports ~4 cases per 100,000 population per year, Texas reports ~3 cases per 100,000 population per year (data from hospitalization records) [26], and Utah reports ~2 cases per 100,000 population per year [27]. Two counties in New Mexico—McKinley and Hidalgo—reported a mean annual incidence of ≥10 cases per 100,000 population per year, nearing the state incidence rate in California.

Of note, in the case time series, there was a sharp decrease in cases observed from 2019 to 2021; this could have been driven by changes in healthcare-seeking behaviors and the transitions of public health resources in response to the COVID-19 pandemic. Similar trends in underdiagnosing and underreporting during the COVID-19 pandemic were observed for coccidioidomycosis throughout the US [28] and other notifiable infectious diseases globally [29,30].

Evident from the high percentage of New Mexico case-patients that were admitted to the hospital (69%), coccidioidomycosis underreporting and underdiagnosing are likely occurring in New Mexico. This is further supported by the high case fatality rate in New Mexico (6.4 deaths per 100 cases) compared with the highly endemic state of Arizona (0.4 deaths per 100 cases [31]. This agrees with a recent spatiotemporal model of coccidioidomycosis cases across the southwestern US, wherein New Mexico had the lowest detection rates of coccidioidomycosis, or the lowest chance that, given there is a coccidioidomycosis case, it will be reported [32]. If New Mexico had the same state-level coccidioidomycosis incidence as Arizona (135 cases per 100,000 population per year [25]), we would expect approximately 2820 cases to be reported in New Mexico each year. However, fewer than 275 cases are reported annually (Figure 1a). Surprisingly, the median time from illness onset to diagnosis in New Mexico (10 days) was less than estimates reported in Arizona (55 days) [33] and California (23 days) [34]. This may be a result of detection bias: only the most severe cases are likely to seek care and receive a more comprehensive diagnostic workup upon presentation.

To provide insights into the current clinical landscape, we encourage a new coccidioidomycosis knowledge, attitudes, and practices survey (KAP). The most recent KAP survey of clinicians licensed in New Mexico (*n* = 425; 14% of all state clinicians) in 2010 found that only 28% were confident in their ability to diagnose coccidioidomycosis, and only 30% were confident in their ability to treat the disease [35]. Similar surveys extended to clinicians in Arizona [36] and veterinarians in Washington [37] have illustrated the need for a comprehensive coccidioidomycosis education campaign. Disease awareness among health officials, hospital professionals, and the public is critical for accurate and swift diagnoses, treatment, and reporting of both humans and companion animals [38].

We found that people who identified as American Indian or Alaskan Native (AI/AN) accounted for 23% of case-patients and had 1.9 times higher incidence rates of coccidioidomycosis than White people (Table 1 and Table 2). This is consistent with prior studies citing that American Indians are at an increased risk for disseminated disease and higher hospitalization rates [5,39,40,41,42]. New Mexico has the fifth-largest AI/AN population in the US (behind Alaska, Oklahoma, Arizona, and California), with 8.6% identifying as AI/AN alone via the US census (mean from 2006–2023) [12]. While there has been a long-standing recognition that people of African [42,43,44,45,46] and Filipino [42,47,48] descent are at greater risk for severe coccidioidomycosis infections, in New Mexico, we did not find significantly higher incidence rates in people who identified as Black or African American or Asian. While the complex underlying host mechanisms for disease vulnerability remain largely undefined [49,50], and socio-economic factors and occupational risks can confound health disparities [51,52], it is important to protect groups that are more vulnerable.

We also observed that older populations in New Mexico are affected more by coccidioidomycosis (Figure 2). This agrees with collective trends observed across 26 states and the District of Columbia, though the incidence among case-patients aged 80 years and older decreased in New Mexico, while nationally, it remains among the age groups with the highest disease incidence [25,26,27]. The coccidioidomycosis incidence in California differs from the national trend and is highest among people aged 40–59 years, which may be driven by work-related or recreational activity exposure to dust [53]. The median age of case-patients in New Mexico was 55 years, older than cases reported in Arizona (median: 51 years) and California (median: 45 years) [25] but younger than those reported in Utah (median: 61 years) [27].

Although coccidioidomycosis is a reportable condition in New Mexico, current NMDOH surveillance is passive, and it relies on a limited set of variables via medical record review. We were unable to make comparisons regarding case-patient work-up, symptoms, comorbidities, treatment, occupation, and travel information due to a lack of data (<200 cases). Conducting enhanced, active surveillance with case interviews would allow for a more accurate history of travel, occupation, hobbies, and other exposure information. This could provide unique information on the coccidioidomycosis risk and exposure in New Mexico. From the few personal interviews available, some locally acquired infections were suspected from puncture wounds and heavy dust inhalation while landscaping [54,55] and working in oil fields in southern New Mexico (e.g., Lea County) [56].

Variations in the number of coccidioidomycosis cases reported each year may suggest that, like other states, environmental conditions drive *Coccidioides* exposure in New Mexico [57,58,59]. Precipitation shapes the seasonal distribution of cases in California and Arizona. In California, there is one peak of precipitation in the winter months, with a subsequent dry season in the summer months, lagging afterward, and coccidioidomycosis cases peak in the fall [60,61]. Arizona experiences a bimodal seasonality of precipitation, receiving rain in the winter and monsoonal storms in the summer. Coccidioidomycosis cases also follow a bimodal trend in the state [62,63]. New Mexico receives most of its precipitation from monsoonal storms (i.e., North American monsoon) from July to September [64]. Precipitation during the non-monsoon months in the state is generally much lower and near constant from month to month. If a similar pattern between precipitation and cases held true for New Mexico, we would expect there to be an increase in coccidioidomycosis cases in the state from winter to spring (Nov.–Mar.). This would allow for *Coccidioides* to grow during the wet monsoon months, followed by the soil drying and *Coccidioides* becoming aerosolized in the following drier autumn and winter months. Though we did not find evidence for a seasonal trend in coccidioidomycosis cases in the time series of case data, we were limited in our statistical power by few case counts, and we likely had additional bias in our dataset due to revisions to the case definitions in 2008, 2011, and 2023 [7,8] (Figure 1c). Understanding the climatic and environmental drivers of coccidioidomycosis dynamics in New Mexico will be crucial for forecasting and projecting disease risk, especially since most of the state is projected to be endemic by 2035 in response to climate change [63,65].

Environmental surveillance throughout New Mexico is critical for identifying populations at risk and targeting locations for enhanced disease surveillance. The results from the soil sampling reported here constitute the first time *Coccidioides* species have been detected in New Mexico soils. While positive samples from prior rodent surveillance suggest Sierra, Catron, and Socorro Counties are likely endemic [6], this study now provides evidence that Doña Ana County is, indeed, locally endemic (Figure 4; Supplementary Table S3; Supplementary Figure S1). Museum collections, specifically frozen tissue archives, offer opportunities for retrospective disease surveillance [66,67]. An insufficient understanding of the ecology of *Coccidioides* species poses challenges in both modeling the disease outbreak potential and forecasting the geographical distribution of the pathogen. An exhaustive list of habitat attributes was investigated (e.g., pH, electrical conductivity, salinity, mineralogy, and vegetation types and density) at *Coccidioides*-positive sites in Arizona, California, and Utah; however, only temperature regimes and soil textures were consistent across all three states [68]. Phenotypic variation among *Coccidioides* species and strains (e.g., thermotolerance [69] and salt tolerance [70]) may affect their suitability for various environments. Ecological niche modeling for *Coccidioides* is likely a matter of scale; thus, environmental detections are necessary for validating and improving these predictions.

The distribution of *Coccidioides* in soils is patchy [71,72,73]; however, *Coccidioides* has been detected at a higher frequency near rodent burrows [74,75,76,77]. This supports the hypothesis that animals may play a vital role in soil enrichment [73,78], which is further evidenced by the ability of species of *Coccidioides* to break down animal proteins [79]. Here, too, we detected *Coccidioides* at a higher frequency in burrows (57.8%) than in topsoil (42.1%), albeit this difference was not statistically significant. Although not part of our study, it has been documented that there is higher rodent activity in grassland sites following dry summers [80]. Considering New Mexican summers have been mostly dry for the last five years [81], there was likely a higher presence of rodents in grassland sites compared with shrublands. Indeed, we had more difficulty locating ten burrows in the mesquite shrubland site. We can only hypothesize about which animals lived in the burrows we sampled. Mammals [82], reptiles [83], amphibians [83], and even fossorial birds [84] may inhabit burrows in New Mexican soils. Our previous work detected *Coccidioides* in otherwise healthy wild animals by leveraging ITS2 fungal metabarcoding of frozen lung tissues from museum collections [6]. This previous study provided evidence that woodrats (*Neotoma albigula* and *Neotoma stephensi*), brush mice (*Peromyscus boylii*), pocket gophers (*Thomomys bottae*), rock squirrels (*Otospermophilus variegatus*), pocket mice (*Chaetodipus intermedius*), and kangaroo rats (*Dipodomys merriami*) are exposed to environmental *Coccidioides* spores in New Mexico (Supplementary Table S3).

Among the three habitat types investigated, we found the most *Coccidioides*-positive samples in grasslands (*n* = 12; 63.2% of positive samples), potentially signaling grasslands as an underrecognized niche for the pathogen. A recent spatiotemporal model mapping endemic areas based on coccidioidomycosis case counts also found grasslands to be positively associated with the likelihood of *Coccidioides* presence, arguing that this association should be further explored [32]. Historically, the presence of *Coccidioides* was anecdotally associated with the presence of mesquite [85,86,87]. Though we did not detect many *Coccidioides*-positive samples in the mesquite-dominated habitat (*n* = 3; 15.8% of positive samples), this could be related to the presence of rodents, as previously mentioned.

A detriment is the lack of whole-genome sequences from New Mexico *Coccidioides* isolates, which would permit in-depth population genetics and functional studies. Currently, a single genome (SRX17215467) is available in NCBI GenBank, but it is absent from metadata. The limited number of sequences is partially due to the multiple barriers that exist for working with *Coccidioides* cultures for whole-genome sequencing, including limited Biosafety Level 3 (BSL3) facilities, extensive training requirements, and difficulty in *Coccidioides* cultivation from soils and non-diseased tissues. It has been postulated that cases endemic to New Mexico will fall within the *C. posadasii* Texas–Mexico–South America clade [88,89], which is plausible given our findings (Figure 4). However, genomic sequences from New Mexico could divulge unique genetic diversity and insights into the evolutionary history of the pathogen. Both nuclear and mitochondrial genomes should be targeted, given that phylogenetic discordance within the group indicates different evolutionary pressures [90].

## 5. Conclusions

Our study brings into question the classification of New Mexico as a low-endemic state for coccidioidomycosis. Based on the disease incidence and evidence for a high amount of underreporting, people in New Mexico are likely among the most at-risk populations for coccidioidomycosis in the US. The highest case incidences were in the Southwest and Northwest regions of the state. American Indian or Alaska Natives (AI/AN) in New Mexico had significantly higher incidence rates of coccidioidomycosis than White people, and older populations in New Mexico had the highest incidence rates. Human cases in New Mexico were caused by both *C. immitis* and *C. posadasii*, but sequence analyses from soils and clinical isolates supported that *C. posadasii* was the main local pathogen.

We report here the first *Coccidioides*-positive soil samples in the state of New Mexico. In combination with previously identified positive rodents, environmental surveillance confirms that locally acquired infections will occur in, but not be limited to, Catron, Doña Ana, Sierra, and Socorro Counties. Our data support previous findings that rodent burrows tend to have a higher positivity rate than surrounding soils, and our results further suggest that grassland habitats may be an underrecognized habitat for the pathogen. Given that seasonal trends for coccidioidomycosis in New Mexico are not yet established, longitudinal soil surveys could offer further insights into endemicity. Both clinical and environmental genomic sequences are necessary to divulge the ecoepidemiology and evolutionary history of Valley fever-causing agents, providing insights into fungal biology, disease ecology, diagnostics, and disease management. We suggest that increased healthcare provider education, epidemiological resources, and environmental monitoring are necessary to ensure timely diagnosis, proper treatment, accurate reporting, and a more granular understanding of distributions in New Mexico. Our findings provide a baseline to inform future research on coccidioidomycosis and *Coccidioides*.

## Figures and Tables

**Table 1:** Demographic and clinical data from coccidioidomycosis cases reported to NMDOH.

Variable		Number (%)
**Case classification**		**1541 (100)**
	Confirmed	946 (61.4)
	Probable	56 (3.6)
	Suspect	539 (35.0)
**Age**		**843 (54.7)**
	Age, y, median (IQR)	55 (40–66)
	Pediatric < 18 y	32 (3.8)
	Geriatric ≥ 65 y	241 (28.6)
**Sex**		**1533 (99.5)**
	Female	653 (42.6)
	Male	880 (57.4)
**Ethnicity**		**581 (37.7)**
	Hispanic or Latino	237 (40.8)
	Non-Hispanic or -Latino	344 (59.2)
**Race**		**854 (55.4)**
	American Indian or Alaska Native	197 (23.1)
	Asian	9 (1.1)
	Black or African American	19 (2.2)
	White	496 (58.1)
	Other race	27 (3.2)
	Multi-race	106 (12.4)
**Outcomes**		**631 (40.9)**
	Hospital admission	438 (69.4)
	Hospital length of stay, d, median (IQR)	6 (3–11)
		**312 (20.2)**
	Mortality	20 (6.4)

**Table 2:** Mean incidence rate ratios for 2006–2023 and 95% confidence intervals of demographics contracting coccidioidomycosis.

Demographic		IRR (95% CI)
**Sex**		
	Female	Referent
	Male	1.6 (1.4–1.7)
**Ethnicity**		
	Non-Hispanic or -Latino	Referent
	Hispanic or Latino	1.3 (0.8–1.8)
**Race**		
	White	Referent
	American Indian or Alaska Native	1.9 (1.4–2.4)
	Asian	0.1 (0.0–0.3)
	Black or African American	1.0 (0.3–1.6)
	Other race	0.6 (0.0–1.2)

**Table 3:** Total number of coccidioidomycosis cases reported, mean annual coccidioidomycosis cases, mean population, and mean annual coccidioidomycosis incidence in 2006–2023 by NMDOH’s Health Jurisdiction.

Jurisdiction		Total Number	Mean Annual Cases Per Year	Mean Population	Mean Annual Incidence (Cases Per 100,000 Population Per Year)
Metro		696	39	897,656	4.3
Northeast		95	5	294,730	1.8
Northwest		228	13	224,919	5.6
	*Northwest* *Tribal*	27	2		
Southeast		136	8	288,132	2.6
Southwest		372	21	369,042	5.6
		**1527**			
Tribal		10	1		
Out of state		2	<1		
**Total**		**1539**			

**Table 4:** Summary of *Coccidioides*-positive soil samples at Jornada Experimental Range in Doña Ana County, New Mexico. We considered samples as positive when at least two of three samples had cycle threshold (CT) values of ≤40. Ecotone is defined here as the transition between grassland and shrubland ecosystems.

Site	Microsite	Positivity
**Grassland**		**12 (12%)**
	Topsoil	6 (12%)
	Burrow	6 (12%)
**Ecotone**		**4 (4%)**
	Topsoil	2 (4%)
	Burrow	2 (4%)
**Shrubland**		**3 (3%)**
	Topsoil	0 (0%)
	Burrow	3 (3%)

## Data Availability

The ITS rRNA sequences from clinical isolates of patients diagnosed in New Mexico are available in GenBank under accessions PQ901548–PQ901562. Alignment and tree files for phylogenetic analyses were deposited in Dryad (https://doi.org/10.5061/dryad.sn02v6xgg). Mitochondrial cox1 sequences from New Mexican soils are available in GenBank under accessions PV177154–PV177156. GPS coordinates for animal surveillance of *Coccidioides* were acquired from the publicly available GitHub repository: https://github.com/p-salazarhamm/Animal_surveillance_Valley_Fever/ (accessed on 12 December 2024).

## Supplementary Materials

The following supporting information can be downloaded at https://www.mdpi.com/article/10.3390/pathogens14060607/s1. Table S1: Metadata of 33 sequences from patients diagnosed with coccidioidomycosis caused by *Coccidioides posadasii* and *Coccidioides immitis* in New Mexico; Table S2: *Coccidioides*-positive soil samples at the Jornada Experimental Range in Doña Ana County, New Mexico; Table S3: Environmental detections of *Coccidioides* in New Mexico; and Figure S1: Mean New Mexico county populations (2006–2023) and health jurisdiction boundaries.
