# Supplementary material for: The Epidemiology of Coccidioidomycosis (Valley fever) and the Disease Ecology of Coccidioides spp. in New Mexico (2006–2023)
Source: Pathogens. 2025 Jun 19;14(6):607. doi: 10.3390/pathogens14060607 (PMC12196052; doi:10.3390/pathogens14060607)
Supplement: Supplementary file 1 [file pathogens-14-00607-s001.zip › pathogens-3599665-supplementary.pdf]

Supplementary Materials

The epidemiology of coccidioidomycosis (Valley fever) and disease ecology of *Coccidioides* spp. in New Mexico

Paris S. Salazar-Hamm[1,2], Sarah Shrum Davis[1,3,4], Jovani Catalán-Dibene[5], Adriana L. Romero-Olivares[5], Karen Edge[3], Andrew W. Bartlow[6], Donald O. Natvig[1], and Morgan E. Gorris[7,*]

[1]Department of Biology, University of New Mexico, Albuquerque, NM 87106 USA
[2]Department of Biology, New Mexico Institute of Mining and Technology, Socorro, NM 87801 USA
[3]Infectious Disease Bureau, New Mexico Department of Health, Santa Fe, NM USA
[4]University of New Mexico Emerging Infections Program, Office for Community Health, Albuquerque, NM USA
[5]Department of Biology, New Mexico State University, Las Cruces, NM 88003 USA
[6]Genomics and Bioanalytics, Los Alamos National Laboratory, Los Alamos, NM 87545 USA
[7]Information Systems and Modeling, Los Alamos National Laboratory, Los Alamos, NM 87545 USA

*Correspondence: mgorris@lanl.gov

This PDF file includes:

Supplemental Tables 1–3

Supplemental Figure 1

Supplemental References

Supplemental Table S1. Metadata of 33 sequences from patients diagnosed with coccidioidomycosis caused by *Coccidioides posadasii* (Cp) and *Coccidioides immitis* (Ci) in New Mexico.

ID	Species	County	State	Race/Ethnicity	Sex	Age	Source/Infection	Occupation	Travel	Significant Past Medical History	Genbank ITS rRNA
**Isolate and patient data from 2018–2023 provided in this study**											
NM5463[a]	Cp	Doña Ana	NM	White/Hispanic	M	60	Pleural fusion/Pulmonary infection	Outdoor construction	Unk	None	PQ901555
NM6180	Cp	Cibola	NM	Unk/Unk	F	43	Tissue/Unk	Unk	AZ	Unk	PQ901550
NM3272	Cp	San Juan	NM	White/Not Hispanic	F	69	Sputum/Pulmonary infection	Unk	Unk	Unk	PQ901551
NM3939	Cp	Unk	NM	Unk/Unk	Unk	Unk	Unk/Unk	Unk	Unk	Unk	PQ901560
NM5410[a]	Cp	Doña Ana	NM	White/Hispanic	M	60	Tissue/Pulmonary infection	Outdoor construction	Unk	None	PQ901554
NM7331	Cp	Hidalgo	NM	White/Hispanic	F	42	Sputum/Pulmonary infection	Unk	Unk	Unk	PQ901552
NM7481	Cp	Eddy	NM	White/Hispanic	M	25	Unk/Unk	Unk	Unk	Unk	PQ901556
NM1900	Ci	Unk	CA	Unk/Unk	M	60	Body Fluid/Unk	Unk	Resident of CA	Unk	PQ901559
NM7039	Cp	Luna	NM	White/Unk	M	36	Bronchial wash/Pulmonary infection	Unk	Unk	Unk	PQ901548
NM4141	Cp	Doña Ana	NM	White/Hispanic	M	42	Sputum/Pulmonary infection	Unk	Unk	Unk	PQ901549
NM6962	Cp	Eddy	NM	Unk/Unk	M	53	Unknown/Unk	Unk	Unk	Unk	PQ901561
NM8365	Cp	Doña Ana	NM	White/Hispanic	F	65	Abcess/Unk	Unk	Unk	Unk	PQ901553
NM8867	Cp	Doña Ana	NM	White/Hispanic	F	58	Sputum/Pulmonary infection	Unk	Unk	Yes, type-II diabetes, smoker	PQ901557
NM9871	Cp	Unk	NM	Unk/Unk	M	88	Sputum/Pulmonary infection	Unk	Unk	Yes, co-infection *Candida krusei*	PQ901558
NM3938	Cp	Lea	NM	White/Hispanic	Unk	53	Sputum/Pulmonary infection	Unk	Unk	Unk	PQ901562
**Isolate and patient data from 2013–2017 previously described [1]**											

NM3006	Ci	San Juan	NM	American Indian	M	60	Bone/Vertebral osteomyelitis	Railroad employee	None	Yes	MH725248
NM9443[b]	Ci	Unk	UT	Unk/Unk	M	51	Ear	Unk	Resident of UT	Unk	MH725258
NM9737[b]	Ci	Unk	UT	Unk/Unk	M	51	Ear	Unk	Resident of UT	Unk	MH725259
NM8725	Cp	Unk	NM	Unk/Unk	Unk	Unk	Fluid/Unknown	Unk	Unk	Unk	MH725256
NM9861	Cp	Bernalillo	NM	Unk/Unk	F	30	Tissue/Pulmonary infection	Unk	None	Yes	MH725261
NM3894	Cp	Bernalillo	NM	Unk/Unk	M	77	Fluid/Knee infection	None	None	None	MH725249
NM0369	Cp	Bernalillo	NM	Unk/Unk	M	66	Bronchial wash/Pulmonary infection	Unk	None	Yes	MH725246
NM4233	Cp	Bernalillo	NM	Unk/Unk	M	48	Tissue/Pulmonary infection	Rug merchant	Tucson, AZ three weeks prior	Yes	MH725251
NM0317	Cp	Chaves	NM	Black/African American	M	62	Unk	Retired. Prior military in CA	None	Yes	MH725245
NM4297	Cp	Eddy	NM	White	M	63	Sputum/Pulmonary infection	Mining engineer	Kansas	Yes	MH725252
NM0071	Cp	Lea	NM	Black/African American	M	28	Sputum/Pulmonary infection	Incarcerated	None	Yes	MH725244
NM3957	Cp	McKinley	NM	Unk/Unk	M	42	Pleural fusion/Pulmonary infection	Unk	Unk	Unk	MH725250
NM9837	Cp	McKinley	NM	American Indian	M	51	Sputum/Pulmonary infection	Welder	Phoenix, AZ	Yes	MH725260
NM5945	Cp	McKinley	NM	American Indian	F	48	Tissue/Pulmonary infection	Unk	None	Yes	MH725254
NM4708	Cp	San Juan	NM	American Indian	F	40	Bronchial wash/Pulmonary infection	Unk	None	Yes	MH725253
NM0459	Cp	Socorro	NM	Unk/Unk	F	50	Tissue/Pulmonary infection	Unk	Unk	None	MH725247
NM7898	Cp	Torrance	NM	Unk/Unk	M	48	Bronchial wash/Pulmonary infection	Incarcerated	None	Unk	MH725255
NM8945	Cp	Unk	AZ	American Indian	M	62	Bone/Osteomyelitis, Pulmonary infection in childhood	Unk	Resident of AZ	Yes	MH725257

[a,b]sequences derived from the same patient

Supplemental Table S2. *Coccidioides* positive soil samples at the Jornada Experimental Range in Doña Ana County, New Mexico. We considered samples as positive when at least two of three samples had cycle threshold (CT) values of ≤40 while reps that were >40 were considered negative (N). A mitochondrial intron sequence that is present in *C. posadasii* (Cp) but not *C. immitis* was amplified by PCR and confirmed by gel electrophoresis. Failure to amplify the fragment was considered not determined (ND) and is likely indicative of low fungal load rather than absence of the intron because all soil samples were taken within 10 km of each other from the grassland dominated sites. Ecotone is defined here as the transition between grassland and shrubland ecosystems.

ID	Site	Microsite	Date	CT rep 1	CT rep 2	CT rep 3	Cp intron amplification (GenBank accession)
C2-T	grassland	topsoil	26-May-2023	26	10	N	
T2-T	ecotone	topsoil	26-May-2023	27	18	N	
M8-B	shrubland	burrow	27-Jun-2023	18	13	N	
M9-B	shrubland	burrow	27-Jun-2023	14	17	N	
C7-B	grassland	burrow	11-Jul-2023	6	3	N	+ (PV177154)
C8-B	grassland	burrow	11-Jul-2023	N	2.5	4.6	ND
C9-B	grassland	burrow	11-Jul-2023	5	4.5	N	ND
C10-B	grassland	burrow	11-Jul-2023	N	3.3	5.2	+ (PV177155)
C1-T	grassland	topsoil	11-Jul-2023	N	6.6	4.5	ND
C4-T	grassland	topsoil	11-Jul-2023	N	3.6	2	
C7-T	grassland	topsoil	11-Jul-2023	1.4	5.5	N	+ (PV177156)
M1-B	shrubland	burrow	11-Jul-2023	3.7	N	4.31	
C2-B	grassland	burrow	25-Jul-2023	N	3.1	3.5	+
C7-B	grassland	burrow	25-Jul-2023	2.9	4.4	N	+
C1-T	grassland	topsoil	25-Jul-2023	5.6	4.1	3.4	ND
C5-T	grassland	topsoil	25-Jul-2023	N	2.4	4.2	ND
T2-B	ecotone	burrow	25-Jul-2023	3.3	25.7	N	
T7-T	ecotone	burrow	25-Jul-2023	7	3.1	N	
T8-B	ecotone	burrow	25-Jul-2023	7	3.1	N	

Supplemental Table S3. Environmental detections of *Coccidioides* in New Mexico.

ID	Date	Sample	Detection Tool	Host	County
**Soil surveillance provided in this study**					
C2-T	26-May-23	topsoil	CocciDx qPCR	N/A	Doña Ana
T2-T	26-May-23	topsoil	CocciDx qPCR	N/A	Doña Ana
M8-B	27-Jun-23	burrow	CocciDx qPCR	N/A	Doña Ana
M9-B	27-Jun-23	burrow	CocciDx qPCR	N/A	Doña Ana
C7-B	11-Jul-23	burrow	CocciDx qPCR	N/A	Doña Ana
C8-B	11-Jul-23	burrow	CocciDx qPCR	N/A	Doña Ana
C9-B	11-Jul-23	burrow	CocciDx qPCR	N/A	Doña Ana
C10-B	11-Jul-23	burrow	CocciDx qPCR	N/A	Doña Ana
C1-T	11-Jul-23	topsoil	CocciDx qPCR	N/A	Doña Ana
C4-T	11-Jul-23	topsoil	CocciDx qPCR	N/A	Doña Ana
C7-T	11-Jul-23	topsoil	CocciDx qPCR	N/A	Doña Ana
M1-B	11-Jul-23	burrow	CocciDx qPCR	N/A	Doña Ana
C2-B	25-Jul-23	burrow	CocciDx qPCR	N/A	Doña Ana
C7-B	25-Jul-23	burrow	CocciDx qPCR	N/A	Doña Ana
C1-T	25-Jul-23	topsoil	CocciDx qPCR	N/A	Doña Ana
C5-T	25-Jul-23	topsoil	CocciDx qPCR	N/A	Doña Ana
T2-B	25-Jul-23	burrow	CocciDx qPCR	N/A	Doña Ana
T8-B	25-Jul-23	burrow	CocciDx qPCR	N/A	Doña Ana
T7-T	25-Jul-23	topsoil	CocciDx qPCR	N/A	Doña Ana
**Wildlife animal surveillance previously reported [2]**					
MSB:Mamm:89752	22-Dec-98	animal	ITS2 fungal metabarcoding	*Neotoma albigula*	Socorro
MSB:Mamm:270443	19-Mar-14	animal	ITS2 fungal metabarcoding	*Peromyscus boylii*	Catron
MSB:Mamm:284485	9-Oct-14	animal	ITS2 fungal metabarcoding	*Thomomys bottae*	Sierra

ID	Date	Sample	Detection Tool	Host	County
MSB:Mamm:303043	13-Oct-17	animal	ITS2 fungal metabarcoding	*Neotoma stephensi*	Sierra
MSB:Mamm:304176	13-Oct-17	animal	ITS2 fungal metabarcoding	*Chaetodipus intermedius*	Sierra
MSB:Mamm:304190	13-Oct-17	animal	ITS2 fungal metabarcoding	*Peromyscus boylii*	Sierra
MSB:Mamm:305238	13-Oct-17	animal	ITS2 fungal metabarcoding	*Neotoma albigula*	Sierra
MSB:Mamm:305243	13-Oct-17	animal	ITS2 fungal metabarcoding	*Neotoma albigula*	Sierra
305246	13-Oct-17	animal	ITS2 fungal metabarcoding	*Otospermophilus variegatus*	Sierra
MSB:Mamm:305240	13-Oct-17	animal	ITS2 fungal metabarcoding	*Chaetodipus intermedius*	Sierra
MSB:Mamm:305241	13-Oct-17	animal	ITS2 fungal metabarcoding	*Dipodomys merriami*	Sierra

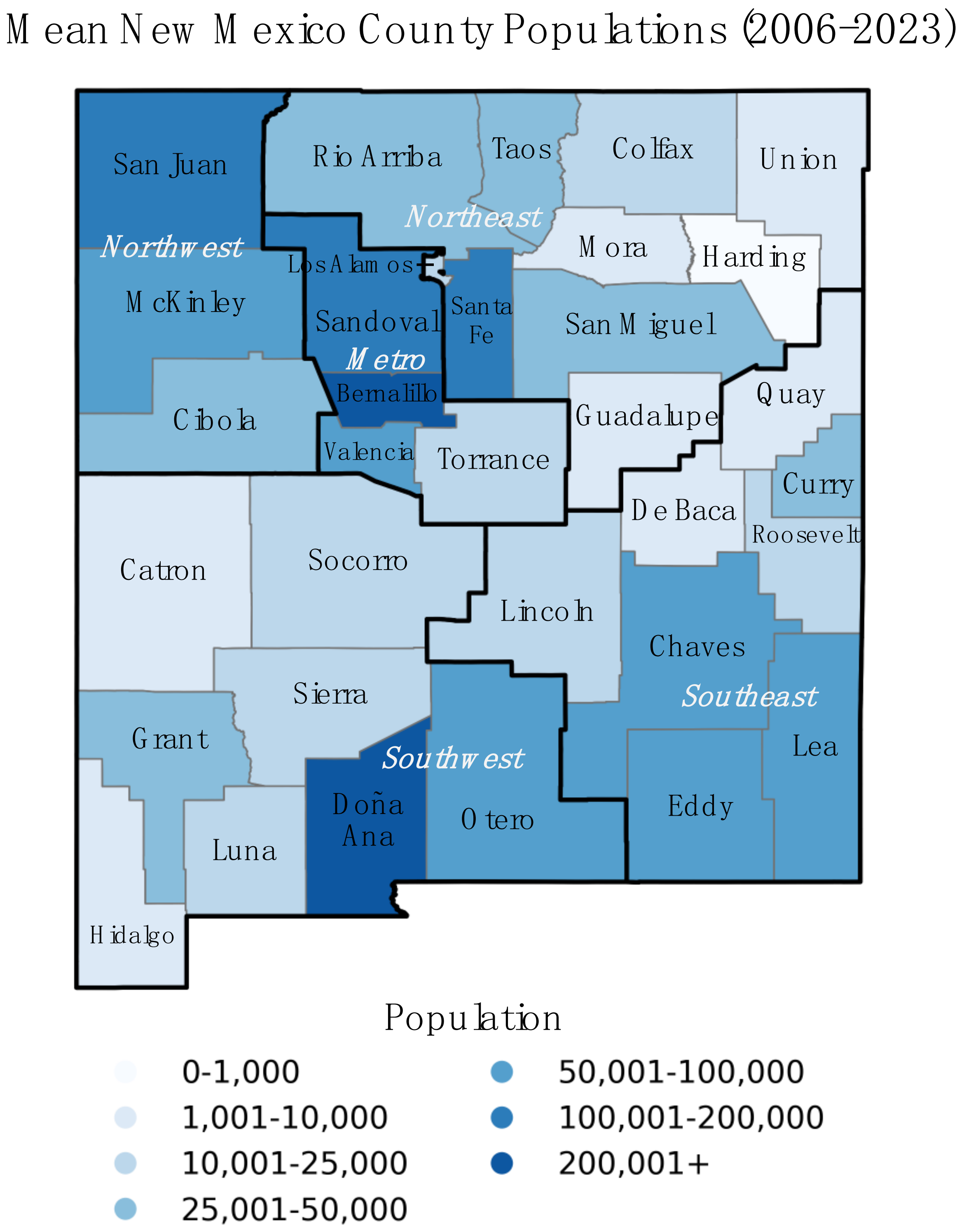

Supplemental Figure S1. Mean New Mexico county populations (2006-2023) and health jurisdiction boundaries. New Mexico county boundaries are shown with gray outlines and county names in black. The five primary health jurisdictions in New Mexico are shown with black outlines and names in white (Metro, Northeast, Northwest, Southeast, and Southwest).

Supplemental References

1. Hamm, P.S.; Hutchison, M.I.; Leonard, P.; Melman, S.; Natvig, D.O. First analysis of human *Coccidioides* Isolates from New Mexico and the southwest four corners region: Implications for the Distributions of *C. posadasii* and *C. immitis* and human groups at risk. *JoF* **2019**, *5*, 74, doi:10.3390/jof5030074.
2. Salazar-Hamm, P.S.; Montoya, K.N.; Montoya, L.; Cook, K.; Liphardt, S.; Taylor, J.W.; Cook, J.A.; Natvig, D.O. Breathing can be dangerous: Opportunistic fungal pathogens and the diverse community of the small mammal lung mycobiome. *Front. Fungal Biol.* **2022**, *3*, 996574, doi:10.3389/ffunb.2022.996574.
